# Optical Coherence Tomography Angiography Analysis of Vessel Density Indices in Early Post-COVID-19 Patients

**DOI:** 10.3389/fmed.2022.927121

**Published:** 2022-06-28

**Authors:** Flavia Chiosi, Giuseppe Campagna, Michele Rinaldi, Gianluigi Manzi, Roberto dell'Omo, Giuseppe Fiorentino, Mario Toro, Fausto Tranfa, Luca D'Andrea, Magdalena Rejdak, Ciro Costagliola

**Affiliations:** ^1^Department of Ophthalmology, Azienda Ospedaliera dei Colli–Ospedale Monaldi, Naples, Italy; ^2^Department of Medical-Surgical Sciences and Translational Medicine, University of Rome “Sapienza”, Rome, Italy; ^3^Department of Ophthalmology, Università della Campania Luigi Vanvitelli, Naples, Italy; ^4^Department of Medicine and Health Science “V. Tiberio”, University of Molise, Campobasso, Italy; ^5^Respiratory semi-intensive Care Unit (UTSIR) COVID, Azienda Ospedaliera dei Colli–D. Cotugno, Naples, Italy; ^6^Department of Ophthalmology, Università degli Studi Federico II, Naples, Italy; ^7^Department of Ophthalmology, University Hospital of Zurich, Zurich, Switzerland

**Keywords:** COVID-19, inflammatory biomarker, OCT angiography, retinal microvasculature, vessel density, summary statement

## Abstract

**Purpose:**

A hypercoagulable state has been reported to cause potential sight-threatening ischemia in patients suffering from Coronavirus disease 2019 (COVID-19). This study aimed to determine whether vessel density (VD), as measured by optical coherence tomography angiography (OCT-A), has insights into retinal and choriocapillaris vascular changes in patients affected by SARS-CoV-2 infection.

**Methods:**

Hundred and fifty two patients positive for SARS-CoV-2 infection were enrolled in this observational, retrospective, controlled study. A control group of 60 healthy subjects was selected for statistical comparisons. Raw OCT and OCT-A data were exported and 3D datasets were analyzed to determine VD.

**Results:**

Hundred and forty eyes (92.1%) were included for final analysis. The VD of the superficial capillary plexus (SCP) did not differ between the two groups. The mean VD of the deep capillary plexus (DCP) and the choriocapillaris (CC) was significantly lower in the foveal sector of the COVID-19 group compared to healthy controls. Within the post-COVID-19 group, the lowest DCP and CC foveal VD values were recorded in patients treated with antiviral therapy; no differences were observed among COVID-19 patients with other comorbidities (hypertension, diabetes, thyroid disease) or taking antiplatelet therapy. DCP and CC foveal VD were significantly lower in patients hospitalized in an intensive care unit (ICU) than asymptomatic patients.

**Conclusion:**

Foveal vessel density at the level of DCP and CC was reduced in post-COVID-19 patients. Further studies evaluating these changes over time will be needed to corroborate the hypothesis of a microvascular retinal impairment in individuals who have recently recovered from SARS-CoV-2 infection.

## Introduction

Coronavirus disease 2019 (COVID-19) is caused by severe acute respiratory syndrome coronavirus 2 (SARS-CoV-2). It is a life-threatening condition primarily affecting the respiratory system but potentially involving all organs including the eye. The ocular surface may serve as a portal of entry for SARS-CoV-2 and can be a source of contagion (1, 2). In fact, SARS-CoV-2 RNA was detected on conjunctival swabs in 0–15% of infected patients using reverse-transcription polymerase chain reaction (RT-PCR) tests (3). Nevertheless, high viral loads in ocular tissue seems to be relatively rare.

Respiratory failure is the most common cause of death in patients infected by SARS-CoV-2, but coagulation activation accompanied by over-production of cytokines, thrombosis, and disseminated intravascular coagulation (DIC) can also lead to a rapid deterioration (4).

Recently, several investigators reported qualitative and quantitative abnormalities of the retinal perfusion in patients with previous COVID-19 infection via both structural optical coherence tomography (OCT) and OCT angiography (OCTA), thus suggesting the occurrence of thrombo-embolic events at the level of the eye (5–7).

However, convincing evidence on retinal and choroid perfusion impairment is still lacking (8). In fact, previous studies mainly focused on analysis of the retinal vessel density (VD) of the superficial and deep retinal capillary plexuses (SCP and DCP) on small samples of patients and reported conflicting results (9, 10). The VD data were limited to the entire macular region with no detailed analysis of the Early Treatment Diabetic Retinopathy Study (ETDRS) subfields and VD measures at the level of the choriocapillaris. Furthermore, the patients differed regarding severity of infection and were visited at different times after resolution of the symptoms; also different detection methods were used (11). These experimental conditions limit the conclusions that can be drawn.

Finally, it remains unclear if factors like severity of the underlying diseases or different therapies may play a role in determining the severity of retinal and choriocapillaris circulation impairment. Thus, the aim of this study was to determine the abnormalities of retinal and choriocapillaris VD as measured by OCTA in a large group of post COVID-19 patients experiencing different grades of disease severity and taking different medications compared to an healthy population.

## Materials and Methods

This non-randomized observational retrospective controlled study was conducted at Azienda Ospedaliera dei Colli-Monaldi and at the affiliated care center for COVID-19, Cotugno Infectious Disease Hospital in Napoli, Italy. All enrolled patients provided written informed consent. This research adhered to the tenets of the Declaration of Helsinki. Ethical approval from the Ethical Committee A.O.R.N. “Ospedali dei Colli” was obtained for this study.

Participants were divided into two groups: the post-COVID-19 group that included 152 eyes from 152 patients who contracted and successively recovered from SARS-CoV-2 infection from June 2020 to December 2020 as well as a control group of 60 eyes from 60 similar age healthy subjects. All clinical data concerning SARS-CoV-2 infection diagnosis and clinical course were obtained from patient records provided by the Infectious Disease Department of the Cotugno Hospital. Inclusion criteria for the post-COVID-19 group were infection testified by two successive oropharyngeal swabs positive in a reverse transcription PCR assay for the SARS-CoV-2 genome. Recovery was confirmed by the simultaneous presence of two consecutive negative swabs, resolution of symptoms (if present) and detection of anti-SARS-CoV-2 IgGs in blood samples.

Patients were classified as follows: (1) asymptomatic, if no symptoms were referred during the complete course of the infection; (2) paucisymptomatic, if mild to moderate symptoms were reported; and (3) symptomatic, if respiratory impairment was recorded (12). All patients underwent a complete clinical anamnesis questionnaire, blood analysis, and lung computerized axial tomography. Inclusion criteria for the control group of healthy patients were two successive oropharyngeal swabs negative for SARS-CoV-2 genome and absence of symptoms suggestive of SARS-CoV-2 infection during the previous months.

Exclusion criteria for both groups were any posterior eye disease that could potentially affect the choroidal or retinal vasculature including high myopia (spherical equivalent ≥6D), exudative age-related macular degeneration (AMD), central serous chorioretinopathy, glaucoma, inherited retinal diseases, diabetic retinopathy, hypertensive retinopathy, optic neuropathy, or previous arterial or venous retinal occlusion.

In the post-COVID group, seven patients were excluded from the study because OCT-A imaging was not gradable, and five presented retinal alterations at the posterior pole. The OCTA images were gradable in 140 eyes (92.1%) on which the final analysis was conducted.

### Scanning Protocol and Quantitative Analysis of OCTA Images

All images were acquired after pupil dilation with 1.0% tropicamide eyedrops. Fundus photography and OCT/OCT-A scans were recorded using the swept source Topcon DRI OCT Triton (Topcon Corporation, Japan). For all subjects, only the eye with the highest image quality scan was used for the analysis. The acquisition protocol consisted of a macular line and 6 × 6 mm macular cube on OCT and OCT-A. All scans were centered on the fovea, and retinal layers were identified using the IMAGENET 6.0 automated layer detection tool to evaluate the superficial capillary plexus (SCPs), deep capillary plexus (DCP), and choriocapillaris (CC). Three-D datasets were analyzed to determine retinal thickness and VD in the five sectors of the Early Treatment Diabetic Retinopathy Study (ETDRS).

Only images with a quality score >50 were deemed acceptable and included in the final analysis. If necessary, multiple attempts were made in order to reach the threshold of image quality at the time of acquisition. The vessel density was defined as the proportion of the total area occupied by the vessels. The OCT-A software, IMAGENET 6.0, was used to generate vessel densities for each scan using the same ETDRS regions as dictated by our study protocol. During this process, the OCT-A signal was extracted and saved for each ETDRS region superimposed on every OCT-A en-face layer. The averaged normalized VD of the full-thickness retina and choriocapillaris of each ETDRS region was then extracted from each participant for further analysis.

### SCPs, DCPs, and CC

The VD was extracted from the retinal nerve fiber layer and ganglion cell layer for the SCP calculation. This analysis moved from the inner plexiform layer and inner nuclear layer for the DCPs and from the retinal pigment epithelium and choroid for the choriocapillaris. The averaged normalized VD from the SCP, DCP, and CC was then extracted for each participant in both groups.

### Outcome Measures

The primary outcome was a difference in the SCP, DCP, and CC VD between the two groups. The following parameters were chosen as secondary outcome measures: central foveal thickness and choroidal thickness. We also performed an additional analysis within the post-COVID-19 group correlating the primary outcome measures with the other variables to reveal potential risk factors for VD impairment in such patients.

### Statistical Analysis

Continuous variables were presented as mean ± standard deviation (SD) and 95% confidence interval (95%CI) or median and (95%CI) and (minimum to maximum). The normality of the continuous variables and residuals was tested using the Shapiro-Wilk test. The categorical variable sex was then compared between the SARS-CoV-2 and control groups using the χ^2^ test and presented as absolute frequencies (*n*) and percentages (%). The Mann-Whitney test was used to compare the age between the SARS-CoV-2 and control groups; the differences between the two groups of the continuous variables measured in superficial capillary plexus (SCPs), deep capillary plexus (DCPs), and choriocapillaris (CC) were performed by Brunner-Munzel test. This test is more powerful than Mann-Whitney test in the presence of ties (12–14).

Student's *t*-test or the Mann-Whitney test, general linear model (GLM), or Kruskal-Wallis test were used to compare the subgroups identified for therapies, underlying diseases, and hospitalization of the of the continuous variables measured in superficial capillary plexus (SCPs), deep capillary plexus (DCPs), and choriocapillaris (CC). Homoscedasticity was evaluated by the Levene and Brown-Forsythe tests. A *p*-value <0.05 was considered statistically detectable. All statistical analyses were performed using SAS v.9.4 (SAS Institute Inc., Cary, NC).

## Results

Demographic and anamnestic data for all subjects are reported in Table 1.

**Table 1:** Demographic and anamnestic Characteristic of patients in the SARS-CoV-2 and Control groups.

**Parameter**	**SARS-CoV-2 mean ± SD; median (min to max)**	**Control mean ± SD; median (min to max)**
Sex^*^ (F/M)	60 (42.85) / 80 (57.14)	27 (45) / 33 (55)
Age (year)	43.27 ± 11.40; 43 (21 to 72)	40.79 ± 12.52; 40 (20 to 65)
Spherical equivalent	21 ± 0.5; (0.5 to 5)	1.75 ± 0.7 (1 to 4.5)
Symptom^*^		
*Asymptomatic*	*29 (20.71)*	
*Paucisymptomatic*	*59 (42.14)*	
*Symptomatic*	*52 (37.14)*	
Lung TAC^*^		
*Negative*	*102 (72.86)*	
*Bilateral pneumonia*	*31 (22.14)*	
*Unilateral pneumonia*	*7 (5.00)*	
Hospital admission^*^		
*No*	*114 (80.85)*	
*Yes*	*27 (19.15)*	
Swab	19.75 ± 10.65; 18 (0.00 to 55)	
Hospital admission days	18.15 ± 10.86; 18 (4 to 51)	
Immunoglobulin (IgG)	31.31 ± 42.13; 10 (0.02 to 170)	

* *Data presented as n (%)*.

Patients in the study group were recruited 1 month after recovery from SARS-CoV-2 infection.

No significant difference was observed in age, sex, intraocular pressure (IOP), or prevalence of myopia between the study group and the control group. Mean spherical equivalent refraction was two (SD ± 1.5; range 0.5 to 5) and 1.75 (SD ± 0.7; range 1 to 4.5) in the post-COVID and control group respectively. The mean age in the post-COVID group was 43.2 ± 11.4 years and 56.3% were male. Here, 20.7% were asymptomatic, 42.2% were paucisymptomatic, and 37.1% were symptomatic. Lung computerized axial tomography revealed bilateral pneumonia in 22.1%, unilateral pneumonia in 5%, and no pneumonia in 72.8% of the cases. In the post-COVID group, 19.15% of the patients were hospitalized because of respiratory impairment while 80.85% did not require hospitalization. The mean duration of hospital admission was 18.15 (±5.6) days. The prevalence of diabetes, hypertension, and thyroid disease in the study group was 0.18, 16.43, and 8.57%, respectively. No significant differences were detected between the study and the control group in terms of incidence of diabetes, systemic hypertension, autoimmune disease, or inflammatory disease. Some patients had Hashimoto thyroiditis (seven in the post-COVID and three in the healthy group). Basedow disease was found in six post-COVID subjects and four control subjects.

Medical therapy was administered as follows: 19.28% were treated with hydroxychloroquine, 28.57% with anticoagulant therapy, 57.14% with azithromycin, 5% with lopinavir and ritonavir, 5% with remdesivir, and 26.42% with corticosteroid therapy.

### VD Measurements: SCP, DCP Segment, and Choriocapillaris Segment

The mean VD derived from the SCP in the five ETDRS sectors did not differ between the two groups (post-COVID vs. control) [16.20 ± 4.04 (95%CI: 15.39 to 17.01) vs. 16.94 ± 2.95 (95%CI: 14.95 to 17.09), *p* = 0.20)]. Conversely, the mean VD of the foveal ETDRS sector recorded in the DCP and CC of the post-COVID group was lower compared to controls (Tables 2, 3).

**Table 2:** Differences of the vessel density between control and SARS-CoV-2 groups in deep capillary plexus (SCPs).

**Parameter**	***Control* mean±SD (95%CI)**	***SARS-CoV-2* mean±SD (95%CI)**	***p***
Central mVD	26.50 ± 3.28 (25.31 to 27.69)	25.53 ± 4.49 (24.72 to 26.34)	**0.008**
Superior	46.86 ± 2.52 (45.82 to 47.90)	47.06 ± 3.31 (46.57 to 47.55)	0.21
Inferior	45.54 ± 2.34 (44.42 to 46.65)	45.11 ± 3.88 (44.52 to 45.69)	0.50
Nasal	44.44 ± 1.53 (43.58 to 45.30)	45.07 ± 2.79 (44.66 to 45.48)	0.07
Temporal	45.34 ± 1.11 (44.49 to 46.19)	45.39 ± 2.84 (44.99 to 45.79)	0.37

*The bold values indicate statistically significant*.

**Table 3:** Differences of the vessel density between control and SARS-CoV-2 groups in choriocapillaris (CCs).

**Parameter**	***Control* mean±SD (95%CI)**	***SARS-CoV-2* mean±SD (95%CI)**	***p***
Central mVD	45.79 ± 5.43 (43.81 to 47.78)	43.09 ± 6.18 (42.28 to 43.90)	**0.006**
Superior	51.11 ± 3.92 (50.08 to 52.15)	51.05 ± 2.65 (50.57 to 51.54)	0.52
Inferior	51.33 ± 1.77 (50.21 to 52.44)	51.11 ± 3.10 (50.52 to 51.69)	0.36
Nasal	49.27 ± 2.38 (48.42 to 50.13)	50.22 ± 2.23 (49.81 to 50.62)	0.10
Temporal	50.66 ± 1.88 (49.81 to 51.51)	51.26 ± 2.09 (50.85 to 51.66)	0.06

*The bold values indicate statistically significant*.

**Figure 1:**
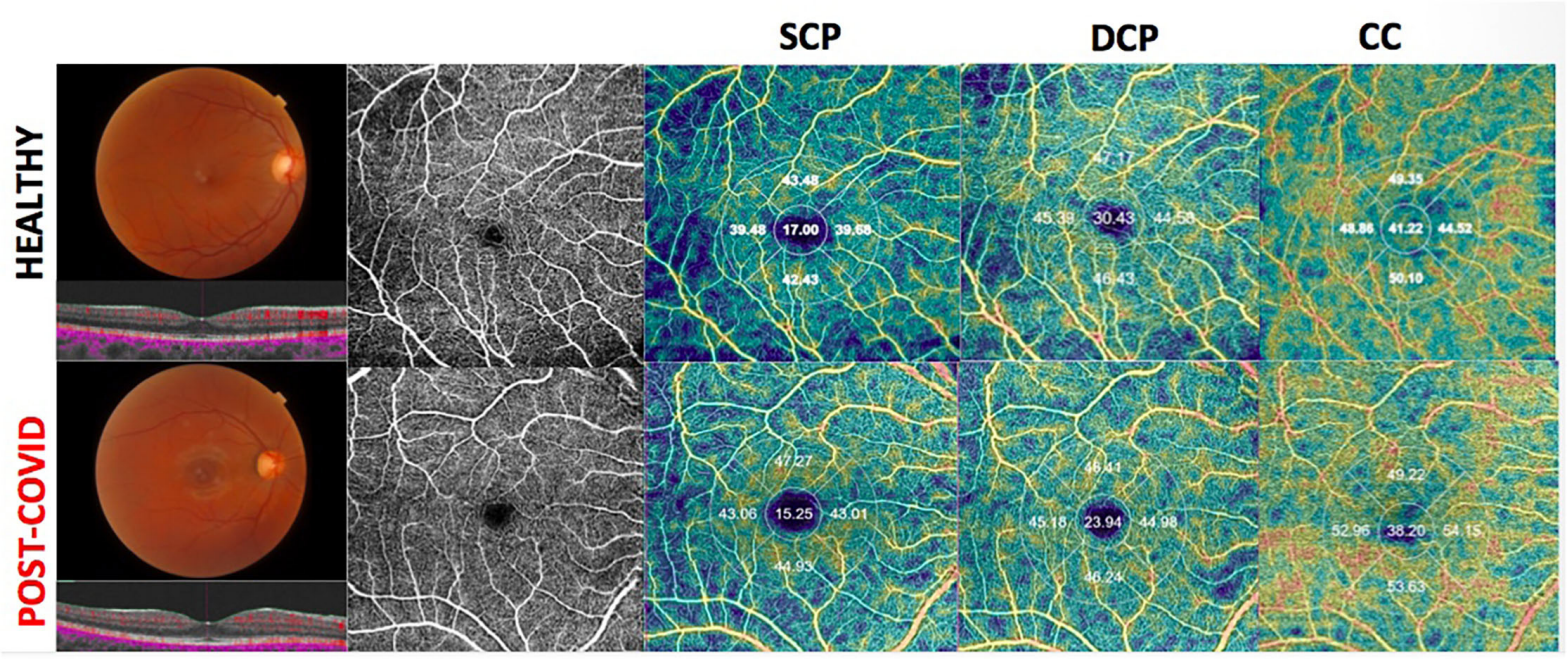
Upper-Right eye of an healthy 41-year-old female shows on Optical Coherence Tomography Angiography (OCTA), a normal vascular texture of the superficial capillary plexus (SCP), the deep capillary plexus (DCP), and choriocapillaris (CC). Bottom-On OCTA the right eye of a post-COVID-19 patient (39- year-old female) reveals a reduced foveal vessel density in DCP and CC with normal SCP indices.

There was no difference for the other ETDRS sectors between the two groups (Figure 1).

### Sub-group Analysis

Within the COVID-19 group, a sub-analysis was performed on the basis of concomitant comorbidities, medications taken by the infected patients, therapies, and severity of the disease. No significant differences were found between patients with hypertension, diabetes, or thyroid disease. Patients treated with antiviral therapy showed a lower mean VD (*p* = 0.017) in the foveal ETDRS sector of the DCP (*p* = 0.017) and CC (*p* = 0.02) compared to patients treated with antiplatelet therapy.

A significantly reduced VD in the foveal ETDRS sector of the DCP (*p* = 0.018) and CC (*p* = 0.014) was recorded in patients admitted to the intensive care unit (ICU) vs. asymptomatic patients.

### Ocular Anterior and Posterior Segment Findings

Ocular anamnestic data were collected from patients during their positivity to the PCR assay for SARS-CoV-2. We observed 12 cases of mild conjunctivitis with no secretion, nine patients complained of epiphora, and 15 reported reduced visual acuity. Myodesopsia and eye strain were recorded in four patients, and three patients reported transitory episodes of diplopia that spontaneously regressed within 2 or 3 days. Other than one patient who developed bilateral focal Candida chorioretinitis during ICU hospitalization, retinal manifestations presented as localized serous detachment of the neurosensory retina at the posterior pole with no visual acuity deterioration in two patients. Two others had sporadic retinal hemorrhages and cotton wool spots. Table 4 shows the ocular findings in our cohort of COVID-19 patients. No correlation was found between ocular posterior or anterior segment alterations and OCTA values.

**Table 4:** Anterior and posterior segment findings in the 152 enrolled patients.

**Ocular findings**	***n* (%)**
Conjunctivitis	12 (7, 8%)
Epiphora	9 (5, 9%)
Visual acuity changes	15 (9, 8%)
Myodesopsia	4 (2, 6%)
Diplopia	3 (1, 9%)
Eye strain	4 (2, 6%)
Chorioretinitis	1 (0, 6%)
Serous retinal detachment	2 (1, 3%)
Retinal hemorrages/CWS	2(1, 3%)

*CWS, cotton wool spots*.

## Discussion

Recent clinical and anatomopathological reports have described endothelial damage as one of the most prominent causes of systemic vascular thromboembolic and/or inflammatory manifestations of COVID-19 (15–17). According to previous studies and our results, the retina is a privileged organ for noninvasive and *in vivo* evaluation of systemic diseases and can reveal these alterations. In fact, we found a reduced foveal VD in the DCP and CC segmentation in 66.6% of patients 1 month after recovery from COVID-19 patients versus the general population.

These findings suggest an impairment in the blood supply to the sub-foveal area of the DCP and choriocapillaris in patients who recovered from SARS-CoV-2. However, these structural changes were not accompanied by visual deterioration.

Recently, other investigators reported on the density of the macular microvasculature and the area of the foveal avascular zone (FAZ) in patients who recovered from COVID-19 using OCT-A (5–7). Our findings differ somewhat from previous studies: Cennamo et al. (6) and Abrishami et al. (5) showed a significant reduction of VD in the SCP and DCP in the whole macular area of the post-COVID patients versus healthy subjects. While Savastano et al. (7) showed unaltered macular and perimacular VD of the SCP and DCP in 70 post-COVID-19 patients. Our results diverge form those of the aforementioned studies, because we detected VD changes only in the foveal sector of the deep and choriocapillaris plexuses, with unaltered SCP. In another study, Abrishami et al. (18). reported data from a three-month follow-up and registered a reduction in the VD and the FAZ area in 18 patients. Although prior work suggested excluding analysis of the FAZ area is deemed to reliably identify changes in vessel density due to the large interindividual variation of this parameter (19). Thus, we decided to not consider FAZ in our analysis. Furthermore, this prior work may have been biased by the substantially lower number of patients recruited (18 patients).

To conduct a more accurate analysis we evaluated the macular area in detail examining data from the five ETDRS macular sectors. This analysis revealed a significant reduction of the VD in the DCP and the CC in our cohort but only in the subfoveal region. Remarkably, none of the previous studies considered choriocapillaris segmentation. We considered this metric to be a valuable measurement of the choriocapillaris VD because choroidal tissue is more likely vulnerable to thrombotic events due to its hemodynamic characteristics; thus, it is likely affected even in absence of a clinically detectable involvement of the other layers. We found that the mean VD of the choriocapillaris at the foveal ETDRS sector was lower than controls and was significantly reduced (along with the VD of the DCP) in patients admitted to the intensive care unit (ICU) compared to asymptomatic patients (*p* 0.034).

In addition, patients treated with lopinavir-ritonavir during admission showed some sign of choriocapillaris VD impairment. These data are consistent with Savastano et al. (20). who observed that the hospitalized patients treated with lopinavir + ritonavir or antiplatelet therapy presented a lower radial peripapillary capillary plexus flow index (RCP-FI) and perfusion density (RCP-PD) due to the endothelial damage induced by such medications. However, several studies have reported that the use of lopinavir-ritonavir does not appear to directly cause endothelial cell dysfunction unlike the dramatic impairment of the circulation caused by indinavir (21). In contrast with the results obtained by Savastano et al. (19), no correlation was found in our study between the use of antiplatelet therapy and VD impairment.

These findings drove us to hypothesize a correlation between VD impairment and the viral coagulopathy associated with COVID-19 especially in patients affected by severe illness such as those requiring antiviral therapy or ICU recovery. Indeed, recent clinicopathologic reports have demonstrated high associations between severe COVID-19 and viral coagulopathy leading to multi-district massive endothelial damage and generalized vascular manifestations (22). Our results seem to support the hypothesis of a positive correlation between a higher viral coagulopathy and outcomes such as ICU stay or VD impairment.

We found that the presence of common systemic co-morbidities such as hypertension, diabetes, or thyroid disease does not seem to influence the severity of the retinal microvasculopathy triggered by SARS-CoV-2 infection. These co-existing conditions do not seem to put these subjects at higher risk of retinal complication or visual loss.

We acknowledge some limitations of this study. Firstly, the small number of healthy control subjects compared to post-COVID-19 patients, which leads to an asymmetry in the sample sizes of the two groups. Therefore, a larger cohort of healthy controls is needed to increase the power of the study. Second, we did not perform OCTA at the time of acute infection, and thus it is possible that we missed some transient retinal or CC vascular impairment. However, several patients experienced severe illness, and it was not possible to acquire images at the time of acute infection. Third, although we demonstrated statistically significant microvascular differences between the post-COVID-19 and the control group, the clinical relevance of such data remains unclear because none patients in the COVID-19 group had a reduction in visual acuity, visual symptoms, or remarkable ocular examination. Also even if patients with high myopia were excluded from the study, axial length was not measured in such population and this might represent a bias for the OCTA values recorded. Finally, the post-COVID patients were not followed over time, and we do not know if the foveal impairment observed 1 month after the acute infection is permanent or may recover.

The strengths of this study include the large sample size, the inclusion of patients with different disease severities, and detailed analysis of each ETDRS subfield as well as analysis of the VD at the level of SCP, DCP, and CC.

In conclusion, retinal vascular impairment was recorded 1 month after recovery in COVID-19 patients by OCTA at the level of the DCP and choriocapillaris segments.

Such parameters might be non-invasive biomarkers for follow-up during the long-term recovery of post-COVID-19 patients. A prospective study is warranted to determine if such vascular impairment is reversible in these patients.

## Summary Statement

A reduced vessel density, as measured by optical coherence tomography angiography, was found in the foveal sector of deep capillary plexus and choriocapillaris in patients who recently recovered from SARS-CoV-2 infection.

## Data Availability Statement

The raw data supporting the conclusions of this article will be made available by the authors, without undue reservation.

## Ethics Statement

The studies involving human participants were reviewed and approved by Comitato Etico - AORN dei Colli. The patients/participants provided their written informed consent to participate in this study.

## Author Contributions

FC: conceptualization and writing. GC: formal and statistical analysis. MRi: data curation. GM and MT: investigation. GF: methodology. Rd'O and CC: review and editing. FT: validation. LD'A: visualization and methodology. MRe: original draft. All authors contributed to the article and approved the submitted version.

## Conflict of Interest

The authors declare that the research was conducted in the absence of any commercial or financial relationships that could be construed as a potential conflict of interest.

## Publisher's Note

All claims expressed in this article are solely those of the authors and do not necessarily represent those of their affiliated organizations, or those of the publisher, the editors and the reviewers. Any product that may be evaluated in this article, or claim that may be made by its manufacturer, is not guaranteed or endorsed by the publisher.

## References

[1] Qu JY Xie HT Zhang MC. Evidence of SARS-CoV-2 transmission through the ocular route. Clin Ophthalmol. (2021) 15:687–96. 10.2147/OPTH.S295283 33658750 PMC7920625

[2] Emparan JPO. Sardi-Correa, C, López-Ulloa JA, Viteri-Soria J, Penniecook JA, Jimenez-Román J, Lansingh VC. COVID-19 and the eye: how much do we really know? A best evidence review. Arq Bras Oftalmol. (2020) 83:250–61. 32490972 10.5935/0004-2749.20200067 PMC11826647

[3] Petronio G Di Marco R Costagliola C. Do ocular fluids represent a transmission route of SARS-CoV-2 infection? Front Med. (2021) 7:620412. 10.3389/fmed.2020.620412 33469546 PMC7813776

[4] Tang N Li D Wang X. Sun Z. Abnormal coagulation parameters are associated with poor prognosis in patients with novel coronavirus pneumonia. J Thromb Haemost. (2020) 18:844–7. 10.1111/jth.14768 32073213 PMC7166509

[5] Abrishami M Emamverdian Z Shoeibi N Omidtabrizi A Daneshvar R Saeidi Rezvani T. Optical coherence tomography angiography analysis of the retina in patients recovered from COVID-19: a case-control study. Can J Ophthalmol. (2021) 56:24–30. 10.1016/j.jcjo.2020.11.006 33249111 PMC7666612

[6] Cennamo G Reibaldi M Montorio D D'Andrea L Fallico M Triassi M. Optical coherence tomography angiography features in post-COVID-19 pneumonia patients: a pilot study. Am J Ophthalmol. (2021) 227:182–90. 10.1016/j.ajo.2021.03.015 33781767 PMC7997850

[7] Savastano MC Gambini G Cozzupoli GM Crincoli E Savastano A De Vico U. Gemelli against COVID-19 Post-Acute Care Study Group. Retinal capillary involvement in early post-COVID-19 patients: a healthy controlled study. Graefes Arch Clin Exp Ophthalmol. (2021) 259:2157–65. 10.1007/s00417-020-05070-3 33523252 PMC7848665

[8] Pirraglia MP Ceccarelli G Cerini A Visioli G d'Ettorre G Mastroianni CM. Retinal involvement and ocular findings in COVID-19 pneumonia patients. Sci Rep. (2020) 10:17419. 10.1038/s41598-020-74446-6 33060700 PMC7566835

[9] Sousa DC Leal I Pinto LA Marques-Neves C. Comment on, optical coherence tomography angiography features in post-COVID-19 pneumonia patients: a pilot study. Am J Ophthalmol. (2021) 227:182–90. 10.1016/j.ajo.2021.08.022 33781767 PMC7997850

[10] Wylegała A Szkodny D Wylegała E. Comment on: optical coherence tomography angiography features in post-COVID-19 pneumonia patients: a pilot study. Am J Ophthalmol. (2021) 234:332. 10.1016/j.ajo.2021.08.021 34619110 PMC8490127

[11] Kelvin YC. Teo, Alessandro Invernizzi, Giovanni Staurenghi, Chui Ming Gemmy Cheung. COVID-19 related retinal micro-vasculopathy - a review of current evidence. Am J Ophthalmol. (2021) 235:98–110. 10.1016/j.ajo.2021.09.019 34587494 PMC8465265

[12] Brunner E. Munzel U. The nonparametric Behrens-Fisher problem: asymptotic theory and a small-sample approximation. Biom J. (2000) 42:17–25. 10.1002/(SICI)1521-4036(200001)42:1&lt;17::AID-BIMJ17&gt;3.0.CO;2-U

[13] Rorden C Karnath HO Bonilha L. Improving lesion–symptom mapping. J Cogn Neurosci. (2007) 19:1081–8. 10.1162/jocn.2007.19.7.1081 17583985

[14] Neuhauser M Ruxton GD. Distribution-Free Two-Sample Comparisons in the Case of Heterogeneous Variances. Behav Ecol Sociobiol. (2009) 63:15. 10.1007/s00265-008-0683-4

[15] Diagnosis of Covid-19. Its Clinical Spectrum. (2020). Available online at: https://www.kaggle.com/einsteindata4u/covid19 (accessed October 26, 2021).

[16] Abou-Ismail MY Diamond A Kapoorc S Arafaha Y Nayak L. The hypercoagulable state in COVID-19: incidence, pathophysiology, and management. Thromb Res. (2020) 194:101–15. 10.1016/j.thromres.2020.06.029 32788101 PMC7305763

[17] Llitjos JF Leclerc M Chochois C Monsallier JM Ramakers M Auvray M. High incidence of venous thromboembolic events in anticoagulated severe COVID-19 patients. J Thromb Haemost. (2020) 18:1743–6. 10.1111/jth.14869 32320517 PMC7264774

[18] Abrishami M Hassanpour K Hosseini S Emamverdian Z Ansari-Astaneh MR Zamani G. Macular vessel density reduction in patients recovered from COVID-19: a longitudinal optical coherence tomography angiography study. Graefes Arch Clin Exp Ophthalmol. (2021) 260:771–9. 10.1007/s00417-021-05429-0 34636996 PMC8505785

[19] Hormel TT Jia Y Jian Y. Plexus-specific retinal vascular anatomy and pathologies as seen by projection-resolved optical coherence tomographic angiography. Prog Retin Eye Res. (2020) 80:100878. 10.1016/j.preteyeres.2020.100878 32712135 PMC7855241

[20] Savastano A Crincoli E Savastano MC Younis S Gambini G De Vico U. Gemelli against COVID-post-acute care study group. Peripapillary retinal vascular involvement in early post-COVID-19 patients. J Clin Med. (2020) 9:2895. 10.3390/jcm9092895 32911619 PMC7565672

[21] Dubé MP Shen C Greenwald M Mather KJ. No impairment of endothelial function or insulin sensitivity with 4 weeks of the HIV protease inhibitors atazanavir or lopinavir-ritonavir in healthy subjects without HIV infection: a placebo-controlled trial. Clin Infect Dis. (2008) 47:567–74. 10.1086/590154 18636958 PMC2919310

[22] Kipshidze N Dangas G White CJ Kipshidze N Siddiqui F Lattimer CR. Viral coagulopathy in patients with COVID-19: treatment and care. Clin Appl Thromb Hemost. (2020) 26:1076029620936776. 10.1177/1076029620936776 32687449 PMC7461127

