# Supplementary material for: Optical Coherence Tomography Angiography Analysis of Vessel Density Indices in Early Post-COVID-19 Patients
Source: Front Med (Lausanne). 2022 Jun 28;9:927121. doi: 10.3389/fmed.2022.927121 (PMC9273855; doi:10.3389/fmed.2022.927121)
Supplement: Supplementary file 1 [file Table_1.doc]

Supplementary Table : Number of patients based on sub-group analysis

| **SCP/DCP/CC** | **n** |
| --- | --- |
| *Antiplatlet therapy* | 16 |
| *Antiviral therapy* | 10 |
| *Asymptomatic* | 21 |
| *ICU-setting* | 19 |
| *Diabetes* | 5 |
| *Thyroid disease* | 12 |
| *Hypertension* | 24 |
